# Cholera Hot-Spots and Contextual Factors in Burundi, Planning for Elimination

**DOI:** 10.3390/tropicalmed6020076

**Published:** 2021-05-11

**Authors:** Amanda K. Debes, Allison M. Shaffer, Thaddee Ndikumana, Iteka Liesse, Eric Ribaira, Clement Djumo, Mohammad Ali, David A. Sack

**Affiliations:** 1 Department of International Health, Johns Hopkins School of Public Health, Baltimore, MD 21205, USA; Allisonshaffer94@gmail.com (A.M.S.); moali.jhsph@gmail.com (M.A.); dsack1@jhu.edu (D.A.S.); 2 Ministry of Public Health, Rue Pierre Ngendandumwe, Bujumbura B.P. 1650, Burundi; ndikumanathaddee@gmail.com (T.N.); iteka.liesse5@gmail.com (I.L.); 3 UNICEF Burundi Country Office, Bujumbura B.P. 1650, Burundi; eribaira@unicef.org (E.R.); cdjumo@unicef.org (C.D.)

**Keywords:** cholera, diarrhea, Burundi, hot spot, oral cholera vaccine

## Abstract

The Republic of Burundi first reported cholera cases in 1978 and outbreaks have been occurring nearly every year since then. From 2008–2020, 6949 cases and 43 deaths were officially reported. To evaluate Burundi’s potential to eliminate cholera, we identified hotspots using cholera incidence and disease persistence as suggested by the Global Task Force for Cholera Control. The mean annual incidence for each district that reported cholera ranged from 0.29 to 563.14 cases per 100,000 population per year from 2014–2020. Ten of 12 Health Districts which recorded cholera cases reported a mean annual incidence ≥5 per 100,000 for this time period. Cholera cases occur during the second half of the year in the areas near Lake Tanganyika and along the Ruzizi River, with the highest risk district being Bujumbura Centre. Additional research is needed to understand the role of Lake Tanganyika; risks associated with fishing; migration patterns; and other factors that may explain cholera’s seasonality. Due to the consistent epidemiological pattern and the relatively small area affected by cholera, control and elimination are feasible with an integrated program of campaigns using oral cholera vaccine over the short term and community-based interventions including WASH activities for sustained control.

## 1. Introduction

### 1.1. Background Information Regarding Burundi

The Republic of Burundi is a landlocked country in the Great Lakes region of Africa which has outbreaks of cholera regularly every year. It lies between Rwanda to the north, Tanzania to the south and east, the Democratic Republic of the Congo (DRC) to the northwest, and Lake Tanganyika to the southwest. The Ruzizi River (also spelled Rusizi River), which flows into Lake Tanganyika from Lake Kivu, serves as the border between the DRC and Burundi. The capital of the country, Bujumbura, is located near the northern part of Lake Tanganyika. The total land area is 27,830 sq km, and the country is divided into 18 provinces which are further divided into 47 districts and 119 communes. The smallest subdivision is the colline, of which there are 2638.

Some of the factors in Burundi that relate to cholera patterns include seasonality, population density, insufficient water-sanitation infrastructure, considerable internal and external migration, poverty, and the relationship of people to Lake Tanganyika.

Burundi has two rainy seasons, a major one from February to May and a lesser one between September and November. The dry seasons are from June to August and December to January. Minimum monthly temperatures appear to be constant throughout the year, but higher maximal temperatures occur from July to October [1].

While the country is geographically small, it is among the top five most densely populated countries in Africa with 463 inhabitants per square kilometers [2]. It has a high fertility rate (about 5.4 children per woman), an infant mortality ratio estimated to be 42 per 1000 live births and a maternal mortality ratio estimated to be 548 per 100,000 live births [2]. Over 90% are employed in agriculture and about 85% of the people are rural subsistence farmers [1], and 65% of the population are living in poverty, or less than USD 1.90 per day per person [3,4].

Indicators of water and sanitation for Burundi demonstrate the need for major improvements. According to the Joint Monitoring Project of the WHO and UNICEF [5], 20% of the population use unimproved or surface water for drinking, and another 20% use water sources that are too far away from their homes. Less than half of the population (46%) has access to basic sanitation, and 40% use unimproved latrines. Handwashing with soap appears to be practiced regularly by only 6% of the population. Thus, many households still rely on unsafe sources for drinking water and lack basic sanitation and hygiene facilities. This is especially true for the lowest quintile and for the rural populations, where the percentages are more worrisome.

Burundi has a long history of both natural disasters and civil conflict, both of which have contributed to its high degree of migration. Within Burundi, people are often displaced because of natural disasters such as landslides, flooding and high winds [6]. When persons are displaced, they are less likely to have access to basic water and hygiene infrastructure. According to a report by the International Organization of Migration (IOM)(7), 90% of 15,000 Internally Displaced People (IDPs) in one area (Bubanza and Kirundo) did not have access to a latrine [7]. Nationally, approximately 42% of collines report that IDP households do not have access to latrines. Further, handwashing systems with soap are not present in 81% of collines, and concerns about the drinking water were reported in 37% of collines. When natural disasters destroy infrastructure such as water sources, roads, etc., people who are not displaced are still affected by the natural disasters and are also at higher risk for cholera. Many displaced persons settle in the provinces along Lake Tanganyika and the Ruzizi River, which our analysis identifies as cholera hotspots.

Migration to and from neighboring countries arise due to violent conflicts, but sometimes are related to the search for employment. As of 31 January 2021, 306,000 people have fled Burundi (almost 3% of the population), with over half of the refugees traveling to Tanzania (148,000) and many others traveling to Rwanda (62,000), the DRC (47,000), and Uganda (50,000) [8]. Since 2017, 125,063 individuals have been repatriated to Burundi as of 31 January 2021, with nearly all of those individuals returning from Tanzania. Returning migrants often have difficulty reintegrating because of the population density, lack of employment and loss of land that they formerly owned. The UNHCR does support their right to return if the decision to return is voluntary, but its stated policy is to not actively promote returning.

Lake Tanganyika plays a critical role in the life and economy of Burundi. The lake is an extremely large, deep, freshwater lake with an alkaline pH (pH 8.5–9); the surface temperature ranges between 25 and 28 °C [9,10] without major differences in characteristics in the water’s physical or chemical properties during the seasons. There have been few publications describing epidemic cholera in Burundi; however, in the late 1990s, *V. cholerae* was isolated from the water of Lake Tanganyika and cholera cases were associated with exposure to or drinking lake water in case control studies [11]. 

### 1.2. Study Background Information

In East and Central Africa, Burundi is one of the countries where cholera is endemic. Burundi first reported cholera cases to the World Health Organization (WHO) in 1978 and cases have been reported in Burundi every year, except during 1986–1989 [12]. Compared to neighboring countries such as the DRC or Tanzania with much larger populations, Burundi reports fewer annual numbers of cholera cases and deaths; still, these outbreaks have a major impact on the country. The reported cases are typically concentrated along Lake Tanganyika, near the border with the DRC. The provinces with cholera cases, unofficially called the “cholera belt”, are usually along the “plain d’Imbo”, which includes the northeast bordering the DRC (Cibitoke, Bubanza) and the provinces along Lake Tanganyika (Rumonge, Makamba, and Bujumbura Mairie/Rural). Interestingly, this pattern has persisted for several decades [11]. In addition to the outbreaks within Burundi, in 2015 a large cholera outbreak occurred in a Burundian refugee settlement in Kigoma, Tanzania, with over 3000 cases and 30 deaths. It is not clear if the refugees from Burundi introduced cholera into the camps or were susceptible because of the conditions in the camps [13].

Cholera cases that were reported to the World Health Organization (WHO) from 2008 through 2018 are shown in Table 1. It should be noted that many countries, especially in Asia, known to have large number of cholera cases do not report or severely under-report cases to the WHO.

Cases of cholera, both suspected and confirmed, are recorded using Burundi’s Integrated Disease Surveillance and Response (IDSR) system [14]. According to the Burundian National Cholera Plan, a “suspected” case, outside of an outbreak, is a patient with acute watery diarrhea, five years or older with severe dehydration, or one who dies from the diarrhea. If a cholera outbreak is ongoing, a suspected cholera patient is one aged five years or older with acute watery diarrhea. During an outbreak, this definition includes diarrhea patients two years or older. A case is “confirmed” when a microbiological culture from the fecal specimen of a suspected case is positive for *Vibrio cholerae* O1 or O139 (Serotype O139 has never been isolated in Burundi). Generally, the National Reference Laboratory in Bujumbura is the laboratory that confirms the case. One case of suspected cholera signifies the alert threshold, while a case must be confirmed before declaring an outbreak, signifying the “action threshold.” The first ten suspected cases are to be cultured, but after one or more are culture-confirmed, it is not necessary to confirm each case.

This epidemiological analysis is intended to provide an overview of the burden of cholera. A principal objective of this analysis was to identify cholera hotspots in Burundi since these are the areas where interventions should focus. A secondary objective was the identification of other factors that may assist in informing intervention and cholera control decisions. Per the GTFCC guidance on developing a national cholera control plan, countries should conduct a situational analysis to include an assessment of the country’s cholera epidemiological situation. This includes the review of historical cholera burden data to identify cholera hotspots and to review contextual factors of hotspots, including population mobility, vulnerability, weather patterns, access to WaSH, among potential other contextual factors [15]. Burundi last updated their National Cholera Control Plan (NCP) in 2012; thus, this analysis may provide information relevant to the National Plan.

## 2. Materials and Methods

### 2.1. Cholera Case Data

Weekly reported suspected cholera case data from Burundi for the years 2014–2020 were obtained from the IDSR surveillance system conducted by the Ministry of Public Health of Burundi (MOPH). Cholera cases are reported at the Health District level. Burundi is divided into 47 Health Districts, and each Health District consists of 2–4 communes. The names of these Health Districts were matched with the names in the reference mapping file. The duration of each outbreak was calculated by comparing the start and end week of reported cases in each Health District. Outbreaks that might have continued across Health District borders were considered as two separate outbreaks.

### 2.2. Population Data

The population data were obtained at the commune level from the most recent census, in 2008. Although the population has increased since then, we assumed this growth was occurring at a similar rate in the different commune [16]. The Health District populations were calculated by adding together the populations of the communes in the Health District.

### 2.3. Geographic Information Systems (GIS) Data

The Health District boundary map of Burundi was obtained from the United Nations Office for the coordination of Human Affairs. The maps were projected in WGS84 UTM coordinates system Zone 37S (http://geokov.com/education/utm.aspx, accessed on 16 February 2021). Data for cholera hotspots were analyzed using the second administrative boundary level as the geographic unit of analysis.

### 2.4. Identification of Hotspots

Hotspots are defined by the GTFCC as a geographically limited area where environmental, cultural and/or socioeconomic conditions facilitate the transmission of the disease in that cholera persists or reappears regularly [15]. We then applied the GTFCC definition which utilizes mean annual incidence and persistence to differentiate low, medium and high-risk districts to identify priority areas/hotspots [17]. 

This analysis used the method for hotspot analysis recommended by the Global Task Force on Cholera Control (GTFCC), which is based on the mean annual incidence of cholera cases and the persistence of the disease [18]. Ideally, this analysis is based on a minimum of five years of weekly or monthly district or administrative level-2 data. As data for more than seven years were available for this analysis, we conducted the analysis to determine if the hotspots are consistent over time, depicting the hotspots for the first 5-year period and compared to the latter 5-year period. Districts were excluded from the analysis if they reported a very low mean annual incidence (less than 5 cases per 100,000 population per year) since these very low rates likely represented falsely reported cases or, if they represented true cases, the low rates suggest the absence of onward transmission.

### 2.5. Definitions in Data Management

The end of a discrete outbreak is defined, per the GTFCC, as the point when two weeks have passed with no further suspected cases [18]. Persistence is measured by the proportion (or percentage) of weeks with cholera being reported in the district. Annual incidence is determined by the total number of cases reported per year divided by the population, the sum of which is multiplied by 100,000 persons. The mean annual incidence is subsequently determined as the average of the time period considered for the analysis (in this case, 5 years). This calculation is further defined in the GTFCC tool [17]. 

Ethical Considerations: As this study only uses secondary, aggregated, de-identified data, the Ministry of Public Health of Burundi determined that ethical approval was unnecessary. Additionally, the IRB at the Johns Hopkins Bloomberg School of Public Health Institutional Review Board determined that this activity was exempt.

## 3. Results

### 3.1. Weekly Number of Cases

Cholera cases were reported every year between 2008 and 2020. Over this 13-year span, 6949 cases and 43 deaths were reported. A more detailed analysis was conducted for two five-year periods for which we had data at district level to facilitate the use of the GTFCC tool. The 7 years of total data were divided into two 5-year periods of analysis, overlapping in years 2016–2018, but facilitating the assessment of whether the hotspots were consistent over time. From 2014 to 2018, 575, 423, 434, 344, and 92 cases were reported for these years, respectively, for a total of 1868 cases and 14 deaths. The second analysis was from 2016–2020, during which 1195 cases and 3 deaths, and 139 cases and 1 death, were reported in 2019 and 2020, respectively. These cases were reported from 12 Health Districts, all on the western side of the country near Lake Tanganyika, or along the Ruzizi River near the border with the DRC.

**Figure 1:**
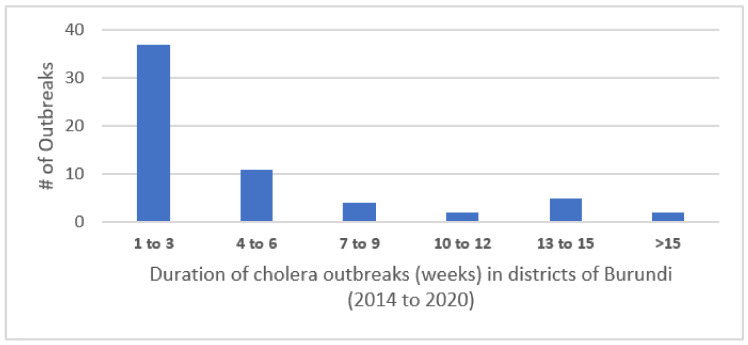
Duration of cholera outbreaks (weeks) in districts of Burundi (2014–2020).

Cases occurred during discrete outbreaks within individual districts experiencing from one to eleven such outbreaks during the 7 years (median of 4.5 outbreaks). The outbreaks lasted from 1 to 24 weeks, with most lasting fewer than 3 weeks. The distribution of the number of outbreaks per district is shown in Figure 1. 

**Figure 2:**
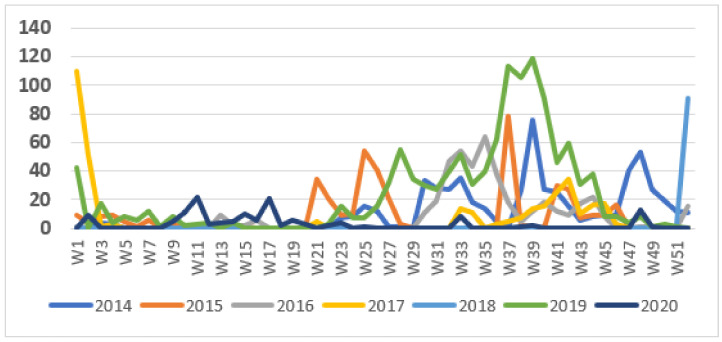
Weekly cases of cholera, 2014–2020.

The weekly number of cases during the surveillance period showed a seasonal pattern, with most cases occurring during the second half of the year (Figure 2). The pattern in 2018 was unusual in that the outbreak started very late in the year, during the last week of 2018, and this outbreak continued into 2019. This was followed by a more typical wave starting in June 2019. Outbreaks tended to start at the end of the dry season but continued into the rainy season.

### 3.2. Hotspot Classification–Mean Annual Incidence and Persistence

**Figure 3:**
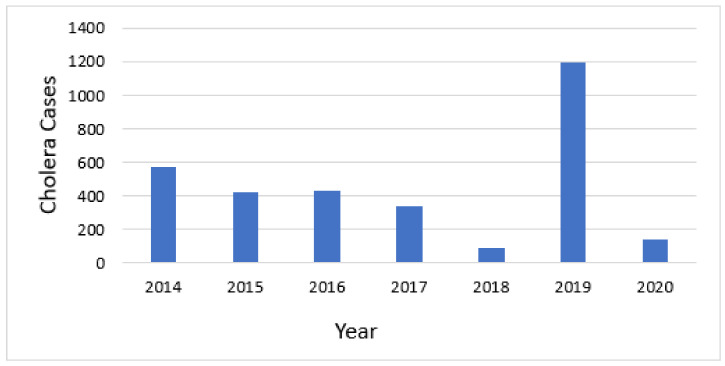
Annual number of cholera cases.

Cholera occurred annually in Burundi during the timeframe of this analysis (Figure 3). The mean annual incidence for each district that reported cholera ranged from 0.29 to 563.14 cases per 100,000 population per year. 

Of 12 Health Districts that reported cholera, ten reported a mean annual incidence ≥5 per 100,000 during the time period of 2014–2020. The cutoff values for determining the magnitude of the cholera risk include applying the median annual incidence (23/100,000) and the median proportion (percentage) of weeks with cholera, defined as persistence (8%), among the nine districts included in the analysis for 2014–2018. For the time period of 2016–2020, the median annual incidence was 13/100,000 and persistence was 5% among the nine districts included in the analysis for this time period. The districts reporting cholera were subsequently grouped into high, medium, and lower risk among the identified hotspots according to the criteria in Table 2.

**Figure 4:**
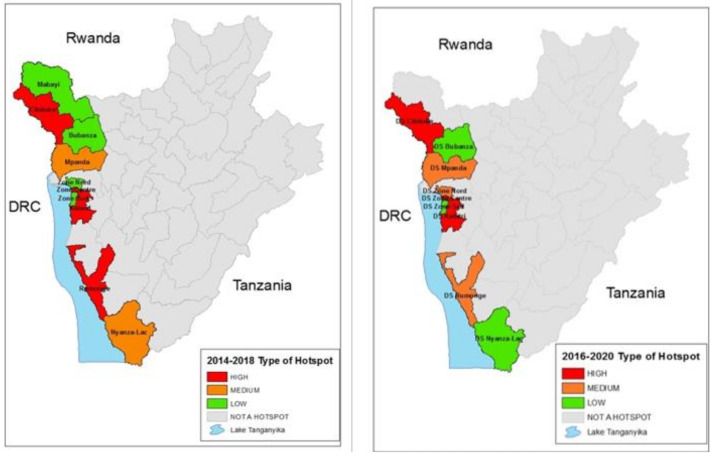
2014–2018 Hotspot classification map as compared to 2016–2020 Hotspot classification map.

When applying the combination of the two cutoffs in Table 2 for the 2014–2018 time period, there were four high-risk districts, one medium-risk district and four low-risk districts. Applying the same cutoffs to the most recent time period of 2016–2020, three health districts were considered as high risk, three were considered medium risk and three were low risk. When applying the cut-offs, other districts in the country were below the threshold for the analysis. The populations of these districts according to the 2008 census are shown in Table 3, and the hotspot districts for the 2014–2018 and 2016–2020 time periods are mapped and shown graphically in Figure 4.

## 4. Discussion

### 4.1. Hotspots and Contextual Factor Findings

This analysis demonstrates that cholera occurred every year in Burundi from 2014 through 2020 and the hotspot pattern proved to be consistent during the period 2014 to 2020. Although there was some variability between the districts in their rank order, the same areas along Lake Tanganyika and along the Ruzizi River, bordering the DRC, were the districts which reported cholera. Other parts of Burundi away from the Lake or the River rarely reported cholera. The designation of high, medium and lower risk hotspots are designations suggested by the GTFCC, and national policy makers will use this information, along with other factors, as they develop plans for cholera control.

Since cholera was first reported in Burundi in 1978, it has remained consistent with very few exceptions. The regular pattern of yearly outbreaks is highlighted further by the striking seasonal pattern in which cholera outbreaks start in the second half of the calendar year and by the consistent locations where the outbreaks occur. The outbreaks occur along the edge of Lake Tanganyika or north of the Lake along the Ruzizi River, which forms the border with the DRC. 

During these outbreaks, several hundred cases are reported. These numbers are smaller than the numbers of cases in neighboring countries with much larger populations, such as the DRC or Tanzania, but they do constitute a great threat to the people of Burundi. Cholera outbreaks require many resources and require the need for very rapid and vigorous response limited health workers to avoid cholera deaths. 

The two time periods of hotspot analysis identified three of the same Health Districts with consistent high priority risk, with a fourth identified in the 2014–2018 time period which remained medium risk in the most recent time period. Both analyses identified additional Health Districts with heightened risk but at a somewhat lower level. The identification of these districts and the predictable nature of the time and place of the outbreaks provide valuable information with which to focus prevention efforts. Such preventive efforts should, of course, include improving water and sanitation infrastructure. For the short term, campaigns to provide oral cholera vaccine to the people living in the highest risk health districts might quickly eliminate these seasonal outbreaks. As the cholera pattern is so consistent, the effectiveness of the OCV campaigns could quickly be evaluated. Being a relatively small country, this would require vaccinating about 1.6 million people living in the districts at risk, or if one were to be more selective, 728,000 living in the highest risk districts identified in either time period. While the maps identify the districts at highest risk, additional local knowledge should be added to provide a more comprehensive understanding of the actual areas at risk. For example, if cases from Bujumbura Centre are actually from the urban area, specific evaluations may be needed to address municipal water and sanitation needs and also consider the specific neighborhoods from which cases arise.

### 4.2. Planning for Elimination

Vaccination campaigns in Burundi would be logistically feasible because the high-risk districts are geographically close to the capital, Bujumbura, where the vaccine could be stored and teams mobilized. The national laboratory is also located in Bujumbura, which simplifies the confirmation of cases following the vaccine campaign; this is important to document the vaccine’s effectiveness.

With financial support from GAVI (Geneva, Switzerland), OCV campaigns have been carried out in several African countries including Malawi [19,20,21], Uganda [22,23], Zambia [24] and South Sudan [25], among others. African countries that have used the largest number of doses include the DRC, Nigeria, Zambia, South Sudan, and Malawi, but 16 African countries have now used OCV [26]. 

OCV is recommended as a two-dose vaccine with an interval of about 2 to 4 weeks between vaccine rounds. Campaigns using a two-week immunization strategy are possible in Burundi; however, cholera’s unique epidemiology in Burundi suggests consideration of other vaccination strategies. Specifically, the first round should be given prior to the expected cholera season (e.g., the month of May). The second round could be given two weeks later, but another option is to wait until the following May to give the second round. The consideration of a delayed second round relates to the protection provided by a single dose [27,28] and the potential that two rounds of vaccine over two years may extend protection because the campaign provided during the second round will immunize additional people who have moved into the area in the intervening year, thus increasing vaccine coverage over a longer period.

In addition to using OCV to prevent cholera, enhanced surveillance would improve the understanding of cholera’s epidemiology in Burundi and would be needed to monitor and evaluate the effectiveness of interventions such as vaccine campaigns and WASH improvements. Rapid diagnostic tests (RDTs) are now available that allow the detection of a case within 15 min. If these were made available in the health facilities in the hotspot districts, outbreaks could be detected rapidly, and samples could be sent to the National Laboratory to declare the outbreak even more quickly. The RDTs can then be used to monitor the course of the outbreak while determining the proportion of cases which are true cases and determine when the outbreak is over. Isolates that are detected at the National Lab should be saved so that molecular testing can confirm their relation to isolates from neighboring countries, especially the DRC and Tanzania.

Reasons for the consistent cholera season in Burundi were not identified. The association of the hotspots being adjacent to Lake Tanganyika and to the border with the DRC suggests the infection may be transmitted from the DRC where cholera is known to be endemic [29]. During the years 2014–2018, over 150,000 cases were reported to the WHO from the DRC, and most of these cases occurred in Eastern DRC. Molecular studies of the strains in the DRC and Burundi would need to be conducted to validate this assumption. Transmission pattern studies and hotspot analyses have been completed in the neighboring country of Uganda [22,30]. Cross-border spread was demonstrated in a study comparing the borders of neighboring countries of Uganda–DRC as well as Malawi–Mozambique, describing how to work to control this risk area [31]. The identification of cholera hotspots near the African Great Lakes is similar to findings from previous studies in the DRC, Uganda, and Malawi [8,21,22,23]. 

Some suggest that a high-risk group are the fishermen who often travel back and forth across the lake. Certainly, fishing villages have been identified as having a higher cholera risk in Uganda [32] and Malawi [33]. The chemistry of lake water is favorable to the maintenance of *V. cholerae*, having an alkaline pH, and *V. cholerae* has been recovered from lake water in the past during an outbreak. If so, it is possible that the domestic use of lake water may be a vehicle of transmission, but this does not explain cholera’s seasonality in Burundi.

The strong seasonality suggests that climate changes may also be related to the timing of outbreaks. During most years, the outbreaks start during the hotter drier months, but then accelerate during the rainy season. It may be that certain climate factors help to initiate an outbreak, but then the rainy season leads to more environmental contamination leading to increased transmission. In endemic areas of Bangladesh and West Bengal, cholera rates have a strong seasonality [34]. 

Safe water and improved sanitation will be critical for cholera control, and certainly the national indicators for water and sanitation demonstrate the need for much improvement. We did not, however, identify specific WASH indicators that would explain the higher risk observed in the hotspot districts. Nevertheless, if RDT and OCV are used in these districts, increased emphasis for improving safe water and sanitation should focus on these districts. The lack of correlation between WASH indicators and cholera risk suggests that data on these risk factors need to be collected from specific subgroups who have had cholera rather than a representative sample of households in the district, most of whom have not had cholera.

### 4.3. Limitations

This analysis had certain limitations. Since this analysis assumes that each district is distinct, the spread of outbreaks between Health Districts is not easily shown and likely under-estimates the true duration of the outbreaks. In fact, many outbreaks did occur simultaneously in different districts and their duration would have extended if the spread between districts was considered. Secondly, only some of the cases were confirmed; however, this method of confirming initial cases and then counting clinically determined cases is consistent with WHO recommendations. Thirdly, we did not have migration histories of the cases to understand where the infection may have been acquired, nor was DNA from Burundian strains available to determine their relation to strains from the DRC or Tanzania. Fourthly, the analysis of hotspots would have benefited if precise GIS points of the actual cases were available. This would have been especially helpful to differentiate urban and rural cases. Fifthly, declaring an outbreak depends on sending samples to the lab in Bujumbura to confirm *V. cholerae*. If rapid tests were used in the field, these might detect outbreaks more quickly. Finally, we used population data from the 2008 census, and we assumed that the increase in numbers would be similar in the different Health Areas. Plans for interventions will need to use updated population numbers when determining the requirements for vaccines and for WASH interventions in these Health Districts. 

## 5. Conclusions

Several factors suggest that cholera could be quickly eliminated as a public health problem in Burundi using an integrated program with OCV for the short term and WASH interventions for the longer term. (1) The country and specifically the hotspot districts are of sufficient size to vaccinate in an organized campaign; (2) the hotspot districts demonstrate consistent cholera persistence over an extended time period; and (3) they are geographically close to the capital of Bujumbura, simplifying logistical requirements for OCV campaigns and follow-up evaluations. 

The GTFCC has set a goal of eliminating cholera from at least 20 countries by 2030, and Burundi should be included on this list. Due to the consistency of cholera patterns, Burundi is unique in that the impact of such an integrated intervention will be apparent very quickly. This epidemiological analysis of Burundi includes the assessment of hotspots and an overview of contextual/risk factors, based on the data available, which may be used in concert with the GTFCC’s framework to update Burundi’s national cholera control plan and plan for elimination [31].

## Figures and Tables

**Table 1:** Number of cholera cases reported to the World Health Organization annually between 2008 and 2018. The totals for the “World” include all cases reported to WHO.

	2008	2009	2010	2011	2012	2013	2014	2015	2016	2017	2018
Burundi	234	355	333	1072	214	1557	582	442	434	300	92
Tanzania	2911	7700	4469	942	286	270	0	11,563	11,360	4895	4777
Uganda	3726	1095	2341	0	6326	748	309	1461	516	292	4440
DRC	30,150	22,899	13,884	21,700	33,661	26,944	22,203	19,182	28,093	56,190	30,768
Rwanda	23	0	0	0	9	0	0	0	355	0	0
All Africa	179,323	217,333	115,106	188,678	117,570	0	105,287	71,176	71,058	179,385	120,652
World	190,130	221,226	317,106	589,854	245,393	129,064	190,549	172,454	132,121	1,227,391	499,447

**Table 2:** Thresholds of mean annual incidence and stability of cases applied per hotspot type.

Type of Hotspots	Mean Annual Incidence Per 100,000 Population (70th percentile value)		Proportion of Weeks with Cholera Reported (60th Percentile Value)	
	2014–2018	2016–2020	2014–2018	2016–2020
High	>23	>13	>8%	>5%
Medium	>23	>13	≤8%	≤5%
Medium	≤23	≤13	>8%	>5%
Lower	≤23	≤13	≤8%	≤5%

**Table 3:** Health Districts categorized by the type of hotspots, based on thresholds of the mean annual incidence and persistence of cholera, defined as the proportion of total weeks during two overlapping time periods.

Type of Hotspot	District	2014–2018		2016–2020		Population (Refers to Groups 2016–2020)
		Mean Annual Incidence/100,000	Proportion of Weeks with Cholera Reported (%)	Mean Annual Incidence/100,000	Proportion of Weeks with Cholera Reported (%)	
High	Bujumbura centre	57.37	16.92	142.12	21.92	123,415
	Cibitoke	30.89	14.23	42.20	16.15	229,867
	Kabezi	37.05	10.38	31.22	6.54	171,665
	Total high					524,947
Medium	Mpanda	15.57	8.08	8.02	5.38	172,138
	Bujumbura nord *	-	-	12.70	3.92	248,915
	Rumonge ^Φ^	29.55	10	18.85	4.62	203,744
	Total Medium					624,797
Lower	Bubanza	6.1	3.1	5.55	1.92	165,885
	Bujumbura sud	5.3	4.2	5.93	4.23	124,836
	Nyanza-Lac ^Ψ^	5.77	4.51	4.51	2.31	203,811
	Mabayi ^δ^	6.2	0.4	-	-	230568
	Total Lower					494,532
Total Hotspots						1,644,276

* Did not appear in earlier analysis but was medium in later analysis. **^Φ^** High in earlier analysis, medium in later analysis. **^Ψ^** Medium in earlier, low in later analysis. **^δ^** Low in earlier analysis, did not appear in later analysis.

## Data Availability

The data presented in this study are available on request from the corresponding author. The data are not publicly available but have been submitted to the GTFCC cholera data collection.
